# Assessing Executive Function in Adolescence: A Scoping Review of Existing Measures and Their Psychometric Robustness

**DOI:** 10.3389/fpsyg.2019.00311

**Published:** 2019-03-01

**Authors:** Moses K. Nyongesa, Derrick Ssewanyana, Agnes M. Mutua, Esther Chongwo, Gaia Scerif, Charles R. J. C. Newton, Amina Abubakar

**Affiliations:** ^1^Neuroassement Group, KEMRI/Wellcome Trust Research Programme, Centre for Geographic Medicine Research (Coast), Kilifi, Kenya; ^2^Utrecht Centre for Child and Adolescent Studies, Utrecht University, Utrecht, Netherlands; ^3^Department of Experimental Psychology, University of Oxford, Oxford, United Kingdom; ^4^Department of Public Health, Pwani University, Kilifi, Kenya; ^5^Department of Psychiatry, University of Oxford, Oxford, United Kingdom; ^6^Institute for Human Development, Aga Khan University Hospital, Nairobi, Kenya

**Keywords:** executive function, assessment, measures, adolescents, scoping review, psychometric properties

## Abstract

**Background:** There is much research examining adolescents' executive function (EF) but there is little information about tools that measure EF, in particular preference of use, their reliability and validity. This information is important as to help both researchers and practitioners select the most relevant and reliable measure of EF to use with adolescents in their context.

**Aims:** We conducted a scoping review to: (a) identify the measures of EF that have been used in studies conducted among adolescents in the past 15 years; (b) identify the most frequently used measures of EF; and (c) establish the psychometric robustness of existing EF measures used with adolescents.

**Methods:** We searched three bibliographic databases (PsycINFO, Ovid Medline, and Web of Science) using key terms “*Adolescents*,” “*Executive Functions*,” and “*measures”*. The search covered research articles published between 1st January 2002 and 31st July 2017.

**Results:** We identified a total of 338 individual measures of EF from 705 eligible studies. The vast majority of these studies (95%) were conducted in high income countries. Of the identified measures, 10 were the most used frequently, with a cumulative percent frequency accounting for nearly half (44%) the frequency of usage of all reported measures of EF. These are: Digit Span (count = 160), Trail Making Test (count = 158), Behavior Rating Inventory of Executive Function (count = 148), Wisconsin Card Sorting Test (count = 140), Verbal Fluency Tasks (count = 88), Stroop Color-Word Test (count = 78), Classical Stroop Task (count = 63), Color-Word Interference Test from Delis-Kaplan battery (count = 62), Rey-Osterrieth Complex Figure Test (count = 62), and Original Continuous Performance Test (count = 58). In terms of paradigms, tasks from Span (count = 235), Stroop (count = 216), Trails (count = 171), Card sorting (count = 166), Continuous performance (count = 99), and Tower (count = 94) paradigms were frequently used. Only 48 studies out of the included 705 reported the reliability and/or validity of measures of EF used with adolescents, but limited to studies in high income countries.

**Conclusion:** We conclude that there is a wide array of measures for assessing EF among adolescents. Ten of these measures are frequently used. However, the evidence of psychometric robustness of measures of EF used with adolescents remains limited to support the validity of their usage across different contexts.

## Introduction

### General Background

Executive function (EF), also known as executive control or cognitive control, is an umbrella term that describes a set of inter-related but distinct cognitive abilities. These cognitive abilities, mediated by the prefrontal cortex (Siddiqui et al., 2008) include, but are not limited to: planning, shifting (i.e., flexibility of thought and action), fluency (i.e., generation of new responses), problem solving, decision making, self-regulation, attentional control, working memory (i.e., concurrent remembering and processing), inhibitory control, and cognitive flexibility (Miyake and Friedman, 2012; Burnett et al., 2013; Costanzo et al., 2013).

Currently, consensus is lacking as to the precise components of EF since it is a multi-faceted construct. Converging research (e.g., Collins and Koechlin, 2012; Lunt et al., 2012; Miyake and Friedman, 2012; Hall and Marteau, 2014; Karbach and Unger, 2014) suggests that EF may be conceptualized best as comprising of three distinct yet related “core” dimensions: working memory, inhibitory control, and cognitive flexibility. Other authors (e.g., Tsermentseli and Poland, 2016; Zimmerman et al., 2016; Poon, 2017) have described EF in terms of “cool” and “hot” components. Cool cognitive skills are elicited under relatively abstract, decontextualized, and non-emotional conditions (Peterson and Welsh, 2014) and require logic and critical analysis (Rubia, 2011). Examples include planning, verbal reasoning, problem-solving, sequencing, cognitive flexibility, working memory, the ability to sustain attention, behavioral monitoring, and inhibition. Hot cognitive skills, in contrast, are elicited in contexts that require personal interpretation where emotions, motivation, or a tension between instant gratification and long-term rewards are generated (Zelazo and Carlson, 2012). Affective cognitive abilities such as social cognition, emotional regulation, affective decision making, and the ability to delay gratification, are posited as aspects of hot EF.

Instead of focusing on individual elements of EF, other investigators in the field have chosen to use theoretical underpinnings on EF for their research purposes. For instance, Burnett et al. (2013), in reviewing the literature on EF and everyday behavior, adopted the Executive Control System conceptual framework (Anderson, 2002; Anderson and Reidy, 2012). This conceptual framework categorizes EF into four broad domains: (i) information processing; (ii) attentional control; (iii) cognitive flexibility; and (iv) goal setting, each consisting of various components tapping into EF. Such a broad categorization, on one hand, overcomes the need of having to focus on components when studying EF. On the other hand, the broadness may lose the precision on the exact construct of EF being studied.

Despite the current lack of consensus about the precise components of EF, it is generally agreed that EF is important for enabling an individual not only to control their emotions and socially interact (Anderson, 2002; Xanthopoulos et al., 2015) but also engage in independent, purposeful, and goal-oriented behavior (Lezak et al., 2004).

### Executive Function in Adolescence

EF skills play an important role in shaping an adolescent's behavior and promoting her/his socio-emotional and educational competencies (Riggs et al., 2006; Bierman et al., 2008). An important aspect of EF is the ability to adaptively respond in circumstances that prime inappropriate and/or prepotent responses, which can lead to impetuous acts or errors in judgment (Prencipe et al., 2011). Adolescence, a period of increasing autonomy, may be of particular vulnerability to such errors partly because EF continues to develop throughout this period (Best and Miller, 2010; Taylor et al., 2015). Moreover, transitioning to adolescence is often followed by a new set of challenging responsibilities and self-regulatory demands for example in educational and social spheres (Burnett et al., 2013) requiring greater reliance on the emerging cognitive control.

It is noteworthy that EF in childhood and adolescence is a predictor of adult productivity and future life outcomes (Diamond, 2013). Therefore, the need to monitor, screen and intervene for EF problems early in life cannot be overemphasized.

### Tools Used to Assess Executive Function at Adolescence

Studying EF in youth populations has received special attention in the recent years, given that it influences their behavioral, social, emotional, and academic outcomes (Arán Filippetti and Richaud, 2015). A wide range of performance-based measures of EF exists and have been used with adolescent sub-population for years. These include tasks such as the Wisconsin Card Sorting Test (WCST; Heaton, 1981); the Trail Making Test (TMT; Reitan, 1992); and the Stroop Color-Word Test (SCWT; Golden and Freshwater, 1978). To assess aspects of EF comprehensively, some performance-based EF tests are administered as a set, in neuropsychological batteries such as the Delis-Kaplan Executive Function System (D-KEFS; Delis et al., 2001), Cambridge Neuropsychological Test Automated Battery (CANTAB; Cambridge Cognition Ltd, 2006), the Behavioral Assessment of the Dysexecutive Syndrome for Children (BADS-C; Emslie et al., 2003), and a developmental Neuropsychological assessment battery (NEPSY; Korkman et al., 1998).

In the new millennium, researchers have also begun to broaden ways of assessing EF among children and adolescents by including self or informant reported questionnaires designed to index children's everyday EF skills. Examples of such EF rating scales include measures such as the Behavior Rating Inventory of Executive Function (BRIEF; Gioia et al., 2000; Guy et al., 2004), the Dysexecutive Questionnaire for Children (DEX-C; Emslie et al., 2003), Amsterdam Executive Function Inventory (AEFI; Van der Elst et al., 2012) and most recently the Dynamic Occupation Assessment of Executive Function (DOAEF; Chubarov et al., 2015).

### Rationale for the Present Study

Despite the existence of a wide array of neuropsychological measures of EF for use with adolescents, little is known about the most preferred (frequently used) measures for this sub-population. Where literature review has been reported, this has been limited to a specific adolescent sub-population such as those living with cerebral palsy (see Pereira et al., 2018). Furthermore, synthesized and summarized information about the psychometric robustness of existing EF measures for use with adolescents i.e., their reliability and ecological validity remains unknown yet this is known among the child (Henry and Bettenay, 2010) and adult population (Pickens et al., 2010). For researchers, neuropsychologists and related practitioners selecting a measure of EF for use in research or clinical purposes, current information on preference, reliability, and validity of EF measures for use with adolescents is essential in helping them make an informed decision.

The aim of this study is therefore to address the above-mentioned knowledge gaps. Specifically, the study examines the following research questions:

1. Which measures are used to assess EF among adolescents within the past 15 years (between January 2002 and July 2017)?
2. Among the identified measures of EF, which are the most preferred or frequently utilized?
3. What is the psychometric robustness of the existing EF measures for use with adolescents?

## Methods

A scoping review was undertaken. Scoping reviews are useful for mapping out and summarizing existing literature on a specific topic in order to assist researchers in identifying the extent, range and nature of the current research evidence (Levac et al., 2010). Our focus was on measures of EF used in studies conducted among adolescents and reported within the last one and a half decade, the aim being to capture the most recent evidence in the field. A methodologically rigorous scoping review framework proposed by Arksey and O'Malley (Arksey and O'Malley, 2005) was applied in the current study. This framework involves five key phases: (i) identifying research question(s); (ii) identifying relevant studies; (iii) study selection; (iv) Extracting and charting the data; (v) collating, summarizing, and reporting the results. Our research questions are listed in the introduction section.

### Identifying Relevant Studies

A search was conducted in three electronic bibliographic databases, that is, PsycINFO, Ovid MEDLINE, and Web of Science. The search terms comprised of the key words “*Adolescents,”* “*Executive Functions,”* and “*measures”* which were combined using the *AND* Boolean operator. Synonyms for each of the key terms were combined using the OR Boolean operator (see Appendix 1 in Supplementary material). The search was limited to only peer reviewed articles, articles in English language, published between 1st January 2002 and 31st July 2017. Where a database could allow, we restricted the search to adolescence age group of interest (13–17 years). Dissertations and book chapters were filtered out.

### Study Eligibility

**Figure 1:**
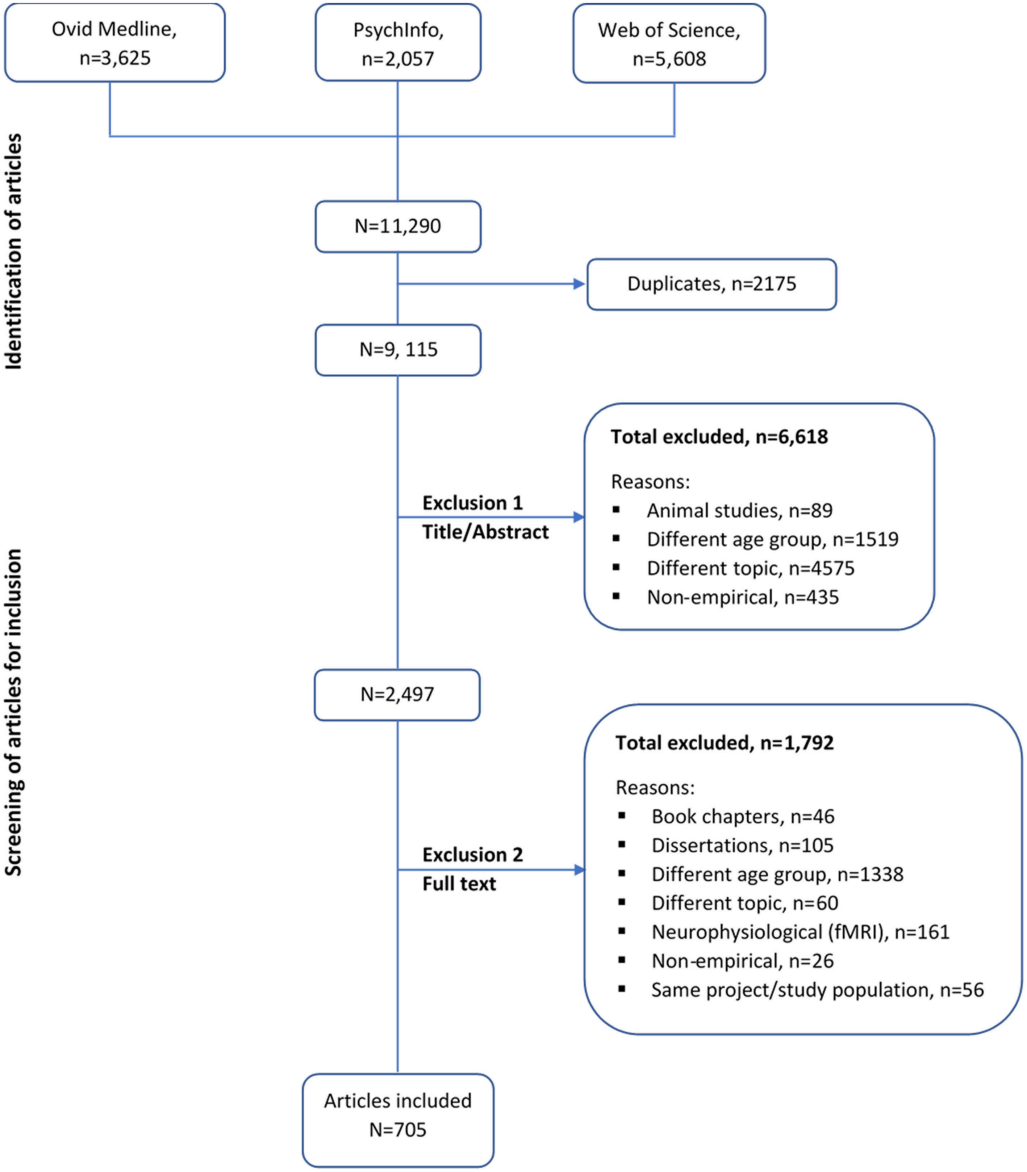
Selection of articles.

Table 1 summarizes the criteria used in the selection of eligible studies for our scoping review. Four authors (MKN, DS, AM, and EC) screened the titles, abstracts, and full-text articles for study eligibility. Where disagreement or doubt arose concerning inclusion or exclusion of an article, the four re-evaluated the article and reached a consensus. Consensus to either include or exclude an article occurred on 25 occasions. Figure 1 illustrates the selection process of the articles.

**Table 1:** Article inclusion and exclusion criteria.

**Criterion**	**Inclusion**	**Exclusion**
Geographical area of interest	Global	None
Target age group	Adolescents 13–17 years, including mean/median, if age range not reported	Age outside 13–17 years
Target EF measure	Neuropsychological measures of EF (tests and rating scales)	Neurophysiological measures of EF e.g., fMRI studies
Type of study	Empirical studies	Non-empirical studies such as systematic reviews/meta-analysis, editorials, case reports
Language of reporting	Articles reported in English	Articles in languages other than English

*fMRI, functional magnetic resonance imaging*.

### Data Extraction

From each study fulfilling the inclusion criteria, information on: author, year, and journal of publication, country in which study was conducted, the measure of EF used in the study and psychometric properties of the measure (if documented) were extracted and charted into Microsoft office Excel (version 2016). For psychometric data, we extracted Cronbach's alpha, intra-class coefficient of correlation (ICC) or any other correlation coefficient, if reported, when documenting the reliability of a given measure. Where a study explored validity of a given measure of EF, we documented the type of validity examined such as construct, content, criterion, concurrent, divergent, or convergent validity, alongside supporting statistics.

### Analysis

We first counted the number of individual measures of EF identified from the review. Then, we computed the frequency of use of each individual measure and summed the frequencies. To describe the most preferred or frequently used measure of EF, we developed a priori working definition, that is, a measure of EF should have accounted for ≥2.5% of the summed frequency of usage of all individual measures of EF (equivalent to a frequency count ≥58 from different included publications). For this analysis, percentage frequencies and cumulative percentage frequencies of measures of EF were computed in Microsoft office Excel (version 2016). We did computations using two approaches. First, we computed frequencies of usage of individual measures of EF (i.e., how frequent an individual measure was used in all the included studies). Second, we grouped measures of EF which we deemed as having a similar underlying principle of measurement (similar underlying latent factor) and computed the frequencies based on this grouping. Data on psychometric properties from the eligible studies were abstracted into a table on Microsoft Office Word (2016). We also coded the countries (and their respective continents) in which the included studies were conducted. These countries were categorized according to World Bank's income ranking (World Bank, 2017). Data were then imported to STATA (version 14.0) statistical software package (StataCorp, 2015) for univariate analyses (frequency and percentage distribution).

## Results

### Measures of EF Used With Adolescents

This scoping review identified 705 eligible studies as shown in the study selection flowchart (Figure 1). From these studies, we identified a total of 338 individual measures that have been used to assess an aspect of EF among adolescents aged 13–17 years (see Appendix 2 in Supplementary material). Most studies used multiple measures and the total frequency count of all the identified individual measures of EF was 2328. Majority of these measures were performance based, with only 13 out of the 338 identified being self- or informant-reported rating scales of EF. Of the 338 measures of EF identified, 10 were most frequently used (Table 2). The cumulative percent frequency of these 10 frequently used individual measures of EF was 43.7%, nearly half of the total frequency count of usage of all identified measures (see Table 2). Appendix 3 in Supplementary material presents a summary of the administration procedures of these 10 most frequent measures of EF.

**Table 2:** Frequency count, percentage and cumulative percent frequency of the frequently used individual measures tapping into adolescents' EF.

**Measure**	**Frequency count**	**%Frequency**	**Cumulative %frequency**
Original Continuous Performance Test (CPT)^*^	58	2.49	2.49
Rey-Osterrieth Complex Figure test (ROCFT)	62	2.66	5.15
D-KEFS Color-Word Interference Test (CWIT)	62	2.66	7.82
Classical Stroop task	63	2.71	10.52
Stroop Color-Word Test (SCWT)	78	3.35	13.87
Verbal fluency tasks	88	3.78	17.65
Wisconsin Card Sorting Test (WCST)	140	6.01	23.67
Behavior Rating Inventory of Executive Function (BRIEF)	148	6.36	30.03
Trail Making Tests (TMT; Part- A and/or B)	158	6.79	36.81
Digit Span (forward and/or backward)	160	6.87	43.69
Other measures of EF^#^	1311	56.31	100.00
**Total**	**2328**	**100.00**	

***D-KEFS**, Delis–Kaplan Executive Functions System (D-KEFS)*.

* The role of attention in EF has been widely debated in the literature. We include Continuous Performance Test as

*a measure of EF because this is how it was classed by authors*.

# *Summed frequency counts of 328 individual measures of EF each with a frequency usage < 2.5%. Only 12 of these were rating scales with Dysexecutive (DEX) questionnaire, Diabetes Related Executive Functioning Scale (DREFS), Decision-Making Quality Scale (count = 2 each) as the most outstanding. The rest of the measures were performance-based, Controlled Oral Word Association Test (COWAT, count = 51) the most outstanding*.

**Figure 2:**
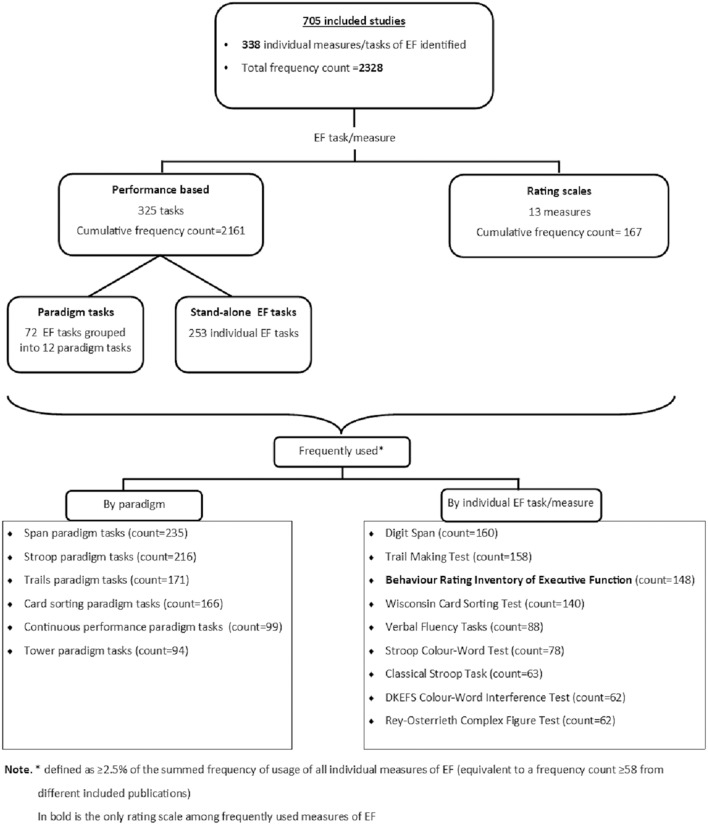
Categorization of identified measures of EF.

Of the 338 individual measures identified, we grouped 72 tasks into 12 paradigms of EF namely Cancellation, Card sorting, Continuous Performance Tests (CPT), Go/No-go, Flanker, Hayling & Brixton, Maze, N-back, Span, Stroop, Tower, and Trails tasks (Table 3). These paradigms consisted of individual measures of EF that assess a common underlying latent factor. The frequency count for these paradigms was 1,116 (47.9% of the total frequency count of all identified measures). Tasks from the Span (count = 235), Stroop (count = 216), Trails (count = 171), Card sorting (count = 166), CPT (count = 99), and Tower (count = 94) paradigms were frequently used. Figure 2 shows how the identified measures of EF were categorized.

**Table 3:** Test variants grouped into paradigms.

**Task variant**	**Frequency**	**%Frequency**
**CANCELLATION TASK PARADIGM**		
Signs Cancellation Tests (2 and 3)	1	12.50
Pair Cancellation test—a non-verbal fluency test	1	12.50
Color Cancelation Test	1	12.50
Number Cancellation test	1	12.50
Bell Cancellation Task	1	12.50
Dot cancellation task	1	12.50
Letter Cancellation Task	2	25.00
Total	8	
**CARD SORTING PARADIGM**		
Dimensional Change Card Sort Test (DCCS)	1	0.60
Wisconsin Monster Sorting Test	1	0.60
Madrid Card-Sorting Test (MCST)	1	0.60
DKEFs Card sorting test	23	13.86
WCST (Wisconsin Card Sorting Test)	140	84.34
Total	166	
**CPT (CONTINUOUS PERFORMANCE TEST) PARADIGM**		
Seidman Continuous Performance Test Vigilance	1	1.01
Integrated Visual and Auditory CPT (IVA)	2	2.02
Conner's CPT	38	38.38
CPT (Original)	58	58.59
Total	99	
**GO/NO-GO PARADIGM**		
Affective Go/No-Go (AGN)	7	13.46
Classical Go/No-go	45	86.54
Total	52	
**FLANKER PARADIGM**		
Flanker Fish Tasks (FF)	1	4.35
Flanker Inhibitory Control and Attention Test	1	4.35
Flanker Shape (FS)	1	4.35
Flanker visual filtering task	1	4.35
Eriksen Flanker Task	19	82.61
Total	23	
**HAYLING AND BRIXTON PARADIGM**		
Brixton Spatial Anticipation Test	2	12.50
Hayling and Brixton tests	4	25.00
Hayling subtest of Hayling and Brixton test	4	25.00
Hayling Sentence Completion Test (HSCT)	6	37.50
Total	16	
**MAZE PARADIGM OF TASKS**		
Executive Maze Task [EM]	1	6.25
Virtual Water Maze	1	6.25
Reasoning and Problem-Solving mazes	1	6.25
Arena Maze	1	6.25
Porteus Mazes [Maze test]	12	75.00
Total	16	
**N-BACK PARADIGM**		
Penn Short Letter N-Back Test (SLNB)	1	5.00
2-n-back task	1	5.00
Spatial n-back	2	10.00
Letter N-back test	3	15.00
N-back test (verbal and/or visual)	13	65.00
Total	20	
**SPAN PARADIGM TASKS**		
Matrix Span Task (MST)	1	0.43
Computation span	1	0.43
Selective span task	1	0.43
Running span task	1	0.43
Spatial memory span task	2	0.85
Recognition span task	2	0.85
Reading Span task	2	0.85
Letter number span	2	0.85
Operation Span Task	2	0.85
Visual Span test	3	1.28
Count span	3	1.28
Word [Problem] Span task	3	1.28
Letter digit span [LDS] task	3	1.28
Category Listening Span (CLS) task	3	1.28
Spatial span task	46	19.57
Digit Span (forward and/or backward)	160	68.09
Total	235	
**STROOP PARADIGM TASKS**		
Stroop Match-to-Sample Task	1	0.46
ClinicaVR: Classroom-Stroop	1	0.46
Motor Stroop task	1	0.46
Emotional Stroop task	2	0.93
Number-quantity Stroop	2	0.93
Chimeric animal Stroop	2	0.93
Counting Stroop task	2	0.93
Stroop residual [interference] test	2	0.93
D-KEFS Color-Word Interference Test (CWIT)	62	28.70
Classical Stroop task	63	29.17
Stroop Color-Word Test (SCWT)	78	36.11
Total	216	
**TOWER PARADIGM**		
Tower of Coimbra	1	1.06
Tower of Hanoi (ToH)	9	9.57
Tower of London (ToL)	41	43.62
DKEFs Tower test	43	45.74
Total	94	
**TRAILS PARADIGM**		
Children's Color Trails Test (CCTT)	2	1.17
Comprehensive Trail Making Test (CTMT)	4	2.34
Color Trails Test (1 and/or 2)	7	4.09
TMT (Trail Making Tests, A and/or B)	158	92.40
Total	171	

***D-KEFS**, Delis-Kaplan Executive Function System*.

A breakdown of the results from the analysis of the regional and income ranking of countries where the EF measures were utilized are presented in Appendix 4 in Supplementary material. In summary, majority of the EF assessments among adolescents aged 13–17 years were conducted either in North America (*n* = 325, 46.1%) or Europe (*n* = 277, 39.3%). Consequently, included studies were mainly conducted in high income countries (*n* = 667, 94.6%) compared to a meager 0.3% representation of low income countries.

### Psychometric Robustness of Identified Measures of EF

Of the 705 included studies in the scoping review, only 48 reported an aspect of reliability and/or validity of a measure of EF used with the adolescent sub-population in a given study setting. These study settings were all high-income countries except for two studies (Wong et al., 2012; Malek et al., 2013) that were conducted in an upper middle-income economic setting of Cuba and Iran. More than half of these 48 studies (*n* = 28) reported the psychometric characteristics of the self- or informant-reported rating scales, with *n* = 22 specifically reporting the psychometrics of the BRIEF (Gioia et al., 2000; Guy et al., 2004; Table 4). Reported internal consistency of the BRIEF ranged from 0.65 to 0.98 in the context of high income countries (both informant- and self-reports; see Table 4 for details). Only one study from United States (Rose and Holmbeck, 2007) reported the inter-rater reliability for the BRIEF (informant report version) which was excellent (0.96 to 0.98). The test-retest reliability for the BRIEF ranged between 0.81 and 0.86. Validity aspects of the BRIEF that were examined across studies included construct, concurrent, and discriminant validity. Presented results, though in the context of high income countries, indicate that BRIEF is a valid measure of EF (see Table 4). It is only in one study from Netherlands (Huizinga and Smidts, 2011), where the Root Mean Square of Error of Approximation (RMSEA) value for the parent-report version of the BRIEF (an estimate of construct validity) was 0.11 which is greater than the recommended value of <0.06 (Thompson, 2004). However, an alternative estimate, Non-Normed Fit Index (NNFI) was excellent at 0.92.

**Table 4:** Reported psychometric properties of EF measures used with adolescents.

**First Author(s) and publication year**	**Country**	**Topic or population studied**	**EF Measure/tool**	**Psychometric characteristic**					
				**Internal consistency** **(α/mean τ)**	**Inter-rater reliability** **(r)**	**Inter informant reliability** **(r)**	**Test–retest reliability** **(ICC)**	**Type of validity examined**	**Validity established?**
Baumgartner et al., 2014	Netherlands	Multitasking and EF in early adolescence	BRIEF-SR (Dutch version)	WM = 0.83; IH = 0.84; CS = 0.72	NR	NA	NR	None	–
Becker and Langberg, 2014	USA	Adolescents with Attention Deficit/Hyperactivity Disorder (ADHD)	BRIEF–PR and TR	BRI = 0.96 (PR); 0.98 (TR) MI = 0.97 (PR); 0.98 (TR)	NR	NR	NR	None	–
Bombin et al., 2013	Spain	Adolescents with Early Onset Psychosis	Stroop Task; TMT WCST	NR	> 0.85 (each)	NA	NR	None	–
Brown et al., 2008	USA	Adolescents with Spina Bifida (SB)	BRIEF-PR	NR	NR	NR	NR	Concurrent	**Yes**. BRIEF and CCT, *r* = −0.23, *p* < 0.05
Burton et al., 2016	USA	Adolescent sexual offenders	BRIEF- SR	BRI = 0.94; MI = 0.95; GEC = 0.97; Subscales = 0.66–0.87	NR	NA	NR	None	–
Byerley and Donders, 2013	USA	Adolescents with Traumatic Brain Injury (TBI)	BRIEF- SR	NR	NR	NA	NR	Construct	**Yes**. SRMSR = 0.03, PNFI > 0.65, RMSEA = 0.05
Carney et al., 2013	UK	Adolescents with Williams and Down syndromes	Listening Span (LS) task Odd-One-Out test (OT) VIMI task Design Fluency (DF)	LS = 0.51 OT = 0.45 VIMI = 0.51–0.67 DF = 0.59–0.83	NR	NA	NR	None	–
Chevignard et al., 2010	Australia	Adolescents who experienced childhood TBI	Children's Cooking Task (CCT)	0.86	NR	NA	0.89	Discriminant	**Yes**. Controls/TBI on CCT *p* < 0.05
								Concurrent	**Yes**. CCT and PRI. *r* = −0.45, *p* < 0.05
Chubarov et al., 2015	Israel	Adolescents with Schizophrenia spectrum disorders	DOAEF	0.83	0.97	NA	0.91	Convergent	**Yes**. DOAEF and WCST, *r* > 0.45, *p* < 0.05
								Discriminant	**Yes**. Group differences, *p* < 0.001
Dougherty et al., 2003	USA	Impulsive behavior and cognitive deficits in an adolescent sample	BVRT	NR	0.97 (NC) 0.97 (NE)	NA	NR	None	–
Duke et al., 2014	USA	Pilot results of psychometric properties of DREFs	DREFS (parent and self-rated)	0.97 (PR) 0.97 (SR)	NR	0.73	NR	None	–
Effeney et al., 2013	Australia	Self-regulated learning and EF in adolescent males	BRIEF-SR	BRI = 0.77 MI = 0.87 GEC = 0.81	NR	NA	NR	None	–
Fino et al., 2014	Italy	EF and impulsivity in general adolescents Adolescents with ADHD or ODD	BIS/BAS	BIS = 0.77 BAS = 0.82	NR	NR	NR	None	–
Forssman et al., 2012	Sweden	Adolescents with ADHD/ Opposition Defiant Disorder (ODD)	Go/No-go task	0.74 (for response inhibition) 0.98 (for response speed)	NR	NA	NR	None	–
Fournet et al., 2015	France	Reliability and Factorial structure of BRIEF (French version)	BRIEF- PR and TR	0.73–0.92	NR	NR	NR	Construct	**Yes**. GFI>0.90 RMSEA/SRMSR < 0.08
Gutiérrez-Colina et al., 2015	USA	Adolescent and Young Adult Transplant Recipients	BRIEF–PR	BRI = 0.93 MI = 0.97 GEC = 0.97	NR	NR	NR	None	–
Hinshaw et al., 2007	USA	Adolescent girls with ADHD	TCFT	NR	NR	NA	0.84 (mean)	None	–
Huizinga and Smidts, 2011	Netherlands	Reliability and Factorial structure of BRIEF (Dutch version)	BRIEF- PR	0.93–0.96 (BRI, MI, GEC)	NR	NR	BRI = 0.84 MI = 0.88 GEC = 0.86	Construct	**Partial**. NNFI = 0.92, RMSEA = **0.11**
Hughes et al., 2009	USA	Adolescents with language impairment	BRIEF-SR and PR	NR	NR	NR	GEC = 0.92	None	–
MacDonald and Duerson, 2015	USA	Reliability of Axon Sports Computerized Cognitive Assessment Tool (CCAT) with high school athletes	CCAT	NR	NR	NA	0.56 −0.67	None	–
Kadish et al., 2013	Germany	Adolescents with epilepsy	EpiTrack Junior®	NR	NR	NA	NR	Concurrent Discriminant	**Yes**. *r* = 0.52, *p* = 0.07 (with WMI), *r* = 0.38, *p* = 0.03 (with PSI) **Yes**. *r* > 0.05 with VLMT
Perez et al., 2016	USA	Adolescents with Type 1 Diabetes	BRIEF—PR	GEC = 0.97 Subscale = 0.83–0.91	NR	NR	NR	None	–
Kirke-Smith et al., 2016	UK	Effect of maltreatment on adolescent EF abilities	Listening Recall (LR) task Odd-One-Out test (OT) VIMI task	LR = 0.78 OT = 0.79 VIMI = 0.89–0.91	NR	NA	NR	None	–
Malek et al., 2013	Iran	Standardization of Stroop Color-Word Test (SCWT) among Iranian bilingual adolescents	SCWT	NR	NR	NA	RT = 0.64–0.93 Err = 0.37–0.81	Discriminant	**Yes**. Significant group differences, *p* < 0.05
Martínez-Loredo et al., 2017	Spain	Reliability and stability of behavioral measures of EF	Stroop Task	RT = 0.85 (wave 1); 0.84 (wave 2) Err = 0.64 (wave 1); 0.68 (wave 2)	NR	NA	NR	None	–
McAuley et al., 2017	Canada	Predictors of persistent Adolescent ADHD	Digit Span Spatial Span	0.85 0.76	NR	NA	NR	None	–
McAuliffe et al., 2008	USA	Adolescents with posttreatment lyme disease	Verbal Fluency Test The Tower Test	0.76 0.48	NR	NA	NR	None	–
Miller and Hinshaw, 2010	USA	Adolescent girls with ADHD	ROCFT	NR	0.90–0.94	NA	NR	None	–
Modi et al., 2017	USA	Adolescents with Epilepsy	BRIEF–SR and PR	0.80–0.98	NR	NR	NR	None	–
Minnes et al., 2016	USA	Adolescents who experienced prenatal cocaine exposure	BRIEF–SR and PR	BRI = 0.96 MI = 0.97	NR	NR	NR	None	–
Na et al., 2013	South Korea	Adolescents with ADHD	Finger Windows Test	0.89	NR	NA	NR	None	–
Owens et al., 2016	USA	Relationship between self-regulation and Sleep Duration, Sleepiness, and Chronotype in adolescents	BRIEF–SR^*^	GEC = 0.85 Subscale = 0.65–0.80	NR	NA	NR	None	–
Park et al., 2016	USA	OCD youths who hoard	BRIEF–PR	0.97	NR	NR	NR	None	–
Perkins et al., 2012	USA	Incarcerated male adolescents	BRIEF–SR	0.71–0.97	NR	NA	NR	None	–
Pesce et al., 2016	Italy	Exploratory evaluation of a life skills program in physical education (PE)	RNG Task	NR	NR	NA	0.70–0.80	Construct	**Yes**. Non- significant X^2^, *p* = 0.77, RMSEA = 0.001 CFI = 0.90
Pope et al., 2016	USA	Association between EF and problematic adolescent driving	BRIEF–SR	0.75–0.98	NR	NA	NR	None	–
Ransom et al., 2016	USA	Adolescents who experienced concussion	BRIEF–SR and PR	NR	NR	NR	NR	Discriminant	**Yes**. Significant group differences, *p* < 0.001
Rose and Holmbeck, 2007	USA	Adolescents with Spina Bifida	BRIEF–PR and TR	NR	0.96–0.98	NR	NR	None	–
Smith et al., 2014	USA	Adolescents with Type 1 Diabetes	BRIEF–PR	0.81–0.98	NR	NR	0.81	None	–
Staiano et al., 2012	USA	Short term effects of an exergame training intervention on adolescent EF skills	Design Fluency TMT	0.97 (Baseline/Treatment) 0.94 (Baseline); 0.98 (Treatment)	NR	NA	NR	None	–
Suchy et al., 2016	USA	Adolescents with Type 1 Diabetes	BRIEF–SR and PR	0.95 (SR) 0.97 (PR)	NR	NR	NR	None	–
Thush et al., 2008	Netherlands	Implicit and explicit alcohol-related cognitions and working memory capacity as predictors of alcohol use after 1 month in at-risk youth	SOPT	0.74	NR	NA	NR	None	–
Van de Weijer-Bergsma et al., 2012	Netherlands	Adolescents with ADHD	BRIEF–PR and TR	0.69–0.95	NR	NR	NR	None	–
Van der Elst et al., 2012	Netherlands	Validation of Amsterdam Executive Function Inventory (AEFI)	AEFI	0.60–0.65	NR	NA	NR	Construct	**Yes**. RMSEA = 0.06, CFI = 0.95, NFI = 0.95
Weiner et al., 2012	USA	Piloting of Weekly Calendar Planning Activity (WCPA) with at-risk adolescents	WCPA	NR	0.99	NA	NR	None	–
Wong et al., 2012	Cuba	Ecological validity of Ballet Executive Scale (BES) for adolescent dancers	BES	0.80	NR	NA	NR	Construct Concurrent	**Yes**. Bifactorial structure of BES: i) SRC (>40% variance); ii) DV (>20% variance) **Yes**. Total score fit LR model
Yoran-Hegesh et al., 2009	Israel	Adolescents with Asperger Disorder	DSST Digit Running Test Stroop Test Simple Reaction Time Choice Reaction Time	NR	NR	NA	0.81 0.71–0.89 0.76–0.95 0.87–0.88 0.79–0.80	None	–
Žebec et al., 2015	Croatia	Inter-relations between processing speed, attention control, working memory, fluid intelligence, and mathematical reasoning from 7 to 18 years of age	Digit Span	NR	NR	NA	0.69 (FDG) 0.66 (BDG)	None	–

*EF, Executive functioning; α, Cronbach's alpha; r, correlation coefficient; ICC, Intra-class correlation coefficient; NR, Not reported; NA, Not applicable; BRIEF, Behavior Rating Inventory of Executive Function; PR, Parent Rated; TR, Teacher Rated; SR, Self Rated; BRI, Behavioral Regulation index; MI, Metacognition index; GEC, Global Executive Composite; WM, Working memory subscale; IH, Inhibit subscale; CS, Cognitive Shifting Subscale; WSCT, Wisconsin Card Sorting Test; TMT, Trail Making Test; VIMI, Verbal Inhibition Motor Inhibition task; PRI, perceptive reasoning index; DOAEF, Dynamic Occupation Assessment of Executive Function; BVRT, Benton Visual Retention Test; NC, Number correct score; NE, Number error score; DREFS, the Diabetes Related Executive Functioning Scale; RMSEA, Root Mean Square of Error of Approximation; SRMSR, Standardized Root Mean square Residual; PNFI, Parsimonious Normed Fit Index. PR, Parent Rated; TR, Teacher Rated; SR, Self Rated; BRI, Behavioral Regulation index; MI, Metacognition index; GEC, Global Executive Composite; BIS/BAS, Behavioral Inhibition System /Behavioral Activation System; TCFT, Taylor Complex Figure Test; GFI, Goodness of fit index; RMSEA, Root Mean Square of Error of Approximation; SRMSR, Standardized Root Mean square Residual Non-Normed Fit Index; NNFI, Non-Normed Fit Index; WMI, Working Memory Index; PSI, Processing Speed Index; VLMT, Verbal learning memory test*.

RT, Reaction Time; Err, Number of errors; NR, Not reported; NA, Not applicable; α, Cronbach's alpha; r, correlation coefficient; ICC, Intra-class correlation coefficient; PR, Parent Rated; TR, Teacher Rated; ^*^, 12 item version; ROCFT, Rey Osterrieth Complex Figure Test; RNG, Random Number Generation Task; X^2^, Chi-square; RMSEA, Root Mean Square error of Approximation.

*TMT, Trail Making Test; SOPT, Self Ordered Pointing Task; DSST, Digit Symbol Substitution Test; FDG, Forward Digit Span; BDG, Backward Digit Span; CFI, Comparative fit index; RMSEA, Root Mean Square of Error of Approximation; NFI, Normed Fit Index; SRC, Self-regulation component; DC, Development component; LR, linear regression*.

The six additional rating scales with reported psychometrics were: the Dynamic Occupation Assessment of Executive Function (DOAEF; Chubarov et al., 2015); Diabetes Related Executive Functioning Scale (DREFS; Duke et al., 2014); Behavioral Inhibition System and Behavioral Activation System (BIS/BAS; Carver and White, 1994); EpiTrack Junior® (Kadish et al., 2013); Amsterdam Executive Function Inventory (AEFI; Van der Elst et al., 2012); and Ballet Executive Scale (BES; Wong et al., 2012). The reported reliability and validity of each of these six measures of EF is presented in Table 4. In summary, their internal consistency ranged between 0.60 and 0.97 (acceptable to excellent); only one study (Chubarov et al., 2015) reported inter-rater reliability and test-retest reliability (for DOAEF) as 0.97 and 0.91, respectively; only one study (Duke et al., 2014) reported inter-informant reliability (for DREFS) as 0.73. Validity aspects that were examined for some of these six measures included construct, convergent, discriminant, and concurrent validities. Presented results indicated that these measures were valid (see Table 4).

Psychometric characteristics of performance based measures, reported from the remaining 20 studies, are shown in Table 4. Unlike for some EF rating scales e.g., the BRIEF, DOAEF, DREFS, and AEFI, where both reliability and validity aspects were reported (Table 4), studies reporting on psychometric characteristics of performance based measures of EF did report either reliability or validity aspect, not both. The exception was in three studies (Chevignard et al., 2010; Malek et al., 2013; Pesce et al., 2016) where both reliability and validity aspects of the children's cooking task (CCT), SCWT, and the random number generation (RNG) task were reported (Table 4). Also, it was only these 3 studies out of the 20 that reported on validity. The discriminant and concurrent validity of the CCT were established in the study by Chevignard et al. (2010); discriminant validity of the SCWT was established in the study by Malek et al. (2013); whereas construct validity of the RNG task was established in the study by Pesce et al. (2016). The performance based measures that were among the 10 frequently used measures of EF (Table 2) had some psychometric characteristics presented (though not extensive and mostly reliability than validity) except for CPT, and D-KEFS Color Word Interference Test (see Table 4 for details). Poor reliabilities were also reported for some complex executive tasks like SCWT (test-retest as low as 0.37) in the study by Malek et al. (2013) and Tower test (internal consistency of 0.48) in the study by McAuliffe et al. (2008).

## Discussion

We carried out a scoping review of measures of EF covering the period between 1st January 2002 and 31st July 2017. We wanted to know three things. First, which measures have been used to assess executive function of adolescents aged 13–17 years. Second, of the identified measures of EF, we were interested in knowing which measures stood out or dominated the field in terms of preference. Lastly, we wanted to establish evidence on the psychometric robustness of measures of EF currently used with the adolescent sub-population.

### Preferred Measures of Adolescent EF

We observed that there is a range of individual EF measures (largely performance based) currently in use with young people aged 13–17 years, although 10 measures of these seem to dominate. Besides, there are a range of individual tasks of EF with a similar underlying principle of measurement (similar latent factor) that have been used to assess EF among adolescents. We grouped these into paradigms to get a better understanding of which group of tasks are frequently selected. We found out that tasks from 12 paradigms were often used and that tasks from the card sorting, CPT, Span, Stroop, Tower and Trails paradigms met our criteria of being frequently used, although in different variations, to assess adolescent EF.

From this review, the most measures of EF currently in use with the adolescent sub-population were performance based. Only 13 out of the 338 identified individual measures of EF were rating scales. This observation was also the same when it came to the 10 dominant measures of EF, where the Behavior Rating Inventory of Executive Function (BRIEF; Gioia et al., 2000; Guy et al., 2004) was the only rating scale. The preference for using performance based measures may reflect either the perceived higher reliability or validity of these measures, or the absence of a wide range of informant/self-report based measures. We prefer the latter explanation for various reasons explained from our findings or elsewhere.

Firstly, from our findings, existing evidence on psychometric robustness of such performance based measures remains scanty (see Table 4 and subheading on psychometric robustness of measures below). Relatedly, the extensive use of experimental paradigms for EF assessment has been criticized because they capture mainly performance at either the pathological or impairment level (Whyte et al., 1996). As a result, most end up having limited functional and ecological validity (Chan et al., 2008). Previous research has also found rating scales such as the BRIEF to be sensitive to changes in executive function even in the absence of changes in performance based measures (Cummings et al., 2002).

Secondly, our findings show that the range of EF ratings scales currently available for use with adolescents is limited. Out of 338 identified measures of EF, only 13 were rating scales. These findings suggest a need for further development or adaptation and validation of measures of EF that are informant/self-report based. Availability of many validated EF rating scales will provide researchers or clinicians a range of options to choose from, but most importantly, enhance the functional and ecological validity of their measurement (Chan et al., 2008). Researchers are already working toward this, if recently developed and validated rating scales such as the Amsterdam Executive Function Inventory (AEFI; Van der Elst et al., 2012), Diabetes Related Executive Function Scale (DREFS; Duke et al., 2014), or Dynamic Occupation Assessment of Executive Function (DOAEF; Chubarov et al., 2015) is anything to go by.

Another reason why researchers and practitioners prefer performance based measures of EF is because some assess multiple components of EF. Focusing on the identified dominant measures, most of the performance based measures tap into more than one aspect of EF. As examples, the Trail Making Test (TMT) assesses domains such as psychomotor speed, cognitive flexibility and working memory (Reitan, 1992) while Wisconsin Card Sorting Test (WCST) is believed to examine aspects such as perseveration, abstract reasoning, working memory and cognitive flexibility (Heaton, 1981). The brief administration time of some performance based measures of EF may also attract some researchers and clinicians in the field. For instance, both the digit span (Blackburn and Benton, 1957) and Stroop Color-Word Test (Golden and Freshwater, 1978) take 5 min or less to administer, whereas the TMT takes 5–12 min (Reitan, 1992).

### Psychometric Robustness of Measures Currently Being Used to Assess Adolescent EF

Only 48 out of 705 studies included in this scoping review reported the reliability and/or validity of a measure of EF used with adolescents. Almost all of the 48 studies were conducted in high income countries, except for two (Wong et al., 2012; Malek et al., 2013) conducted in upper middle-income countries. This is not surprising because in this scoping review, we observed that most of the studies assessing adolescent EF have been conducted in North America and Europe (see Appendix 4 in Supplementary material). These are the same settings in which the majority, if not all, of the existing measures of EF have been developed and validated. Therefore, the reported psychometrics largely reflects performance of EF measures only within a restricted context. For adolescent EF to be accurately assessed across contexts, validation work should be extended to cover low-to-middle income countries where there is hardly any contextually appropriate measure of EF, yet it is in such settings where a great majority of the world's adolescents live in WHO (2014).

The reported psychometrics characteristics were mainly for EF rating scales (28 out of the 48 studies). These rating scales include the BRIEF (Gioia et al., 2000; Guy et al., 2004), DOAEF (Chubarov et al., 2015), DREFS (Duke et al., 2014), AEFI (Van der Elst et al., 2012), EpiTrack Junior® (Kadish et al., 2013), the Ballet Executive Scale (BES; Wong et al., 2012), and Behavioral Inhibition System and Behavioral Activation System (BIS/BAS; Carver and White, 1994). All, except BIS/BAS have been developed and validated within the last decade. The psychometric characteristics of these rating scales, even though from confined context, are good. A step forward in research will be to see more reports of how these tests perform when adapted and used in low-to-middle income countries.

Being one of the dominant measures used to assess adolescent EF, BRIEF predominated in terms of reported psychometrics. Twenty-two out of the 48 studies in this scoping review reported the psychometric characteristics of the BRIEF. Apart from two studies (Burton et al., 2016; Owens et al., 2016) where the reported sub-scale internal consistency of the BRIEF was below the recommended cut-off standard of 0.70 (Cicchetti, 1994), all the other reported reliabilities (Cronbach alphas, inter-rater and test-retest) ranged from good to excellent. BRIEF appears to be a valid measure of EF in terms of its construct, concurrent, and discriminant validity. Generally, these patterns of results re-affirm the good psychometric properties of the BRIEF as originally reported (Gioia et al., 2000; Guy et al., 2004). More work needs to be done from settings other than high income countries to confidently conclude that BRIEF can be validly used across contexts.

Evidence of psychometric robustness of performance based measures, even in the restricted context of upper middle income and high-income countries, remains scanty. Majority of the studies reporting on psychometrics of performance based measures focused on reliability aspect, though not extensively. Limited psychometrics are also presented for the dominant measures of EF we identified, mostly reliability. For some complex executive tasks like the Stroop Color-Word Test in the study by Malek et al. (2013) and Tower test in the study by McAuliffe et al. (2008), poor reliabilities are presented (see Table 4). Such findings mirror the observation that “complex executive tasks tend to suffer from relatively low internal and/or test–retest reliability” (Miyake et al., 2000). A major weakness is noticeable in terms of validity where only 3 studies (Chevignard et al., 2010; Malek et al., 2013; Pesce et al., 2016) reported on an aspect of validity of performance based measures. Among the dominant performance based measures of EF, only the Stroop Color-Word Test (Golden and Freshwater, 1978) has some evidence of validity for use with adolescents. Despite the lack of concrete evidence about psychometric robustness of performance based measures of EF other than backup by original test developers, these measures continue to be a preferred option overtime because of two potential reasons. First, we think that the relatively long history of development and use of performance based measures of EF, such as the Stroop task (Stroop, 1935), provides some degree of confidence in using them. Second, in reference to findings from this review, it could be that because some performance based measures of EF are used in almost similar contexts as to where they were originally developed and tested, researchers rarely focus on exploring their psychometric stability.

In summary, a notable conclusion from this review is the fact that there is just not enough validity and reliability data to support the use of measures of EF among adolescents across different national and cultural contexts. Similar observations are also noted from a recent review (Pereira et al., 2018). Of concern here, therefore, is the transference and use of these measures of EF in different cultural contexts without adequate adaptation and standardization. This can lead to significant limitations of interpreting findings (Greenfield, 1997), constrict the within-group variance or mask true differences between study groups (Grantham-McGregor, 1993). Assessment bias can also arise because of a lack of familiarity with test demands or content (Baddeley et al., 1995; Vijver, 1997). Findings from assessment of adolescent EF especially in low-to-middle income countries using unstandardized measures may not be a true reflection of their EF performance and can misguide policy or intervention efforts. Given the current scenario, it is essential that researchers adapt and/or develop context-sensitive measures of EF that possess adequate psychometric characteristics for use with adolescents.

## Strengths and Limitations of the Review

We chose to conduct a scoping review as it is the most recommended where the nature of research (questions) is anticipated to be large/broader, complex, or highly heterogeneous hence not amenable to a more precise systematic review (Peters et al., 2015). We conducted this scoping review in a systematic and rigorous manner following the recommended scoping review framework (Arksey and O'Malley, 2005). However, the review has some limitations that need to be highlighted. Despite the search strategy being a thorough one, we only searched 3 major electronic databases of MEDLINE, PsycINFO, and Web of Science. Therefore, we cannot be certain that all important data were extracted and consequently reported. We also included and reviewed published articles entirely from journals. We did not search the gray literature. We included articles published in English only because this was the main language that authors are familiar with. We therefore acknowledge that the published literature may not be representative of entirety of work examining EF among adolescents. Because of inconsistency across included studies in reporting the EF domain assessed by tasks/measures of EF we identified, it was difficult to collate information on tasks/measures by EF domain assessed.

## Conclusions

There is a range of measures currently in use to evaluate different aspects of executive function among the adolescent sub-population, although 10 measures appear to dominate the field. Unfortunately, there is very limited evidence generated to support the validity and usage of these measures of EF among adolescents across different national and cultural contexts.

## Author Contributions

AA, CN, and GS conceptualized the study. MN and AA refined the search strategy and conducted the database search. MN, DS, AM, and EC screened and extracted data from all included articles. MN wrote the first draft of the manuscript. DS, AM, EC, GS, CN, and AA all critically reviewed the first draft and subsequent revisions of the manuscripts. All authors read and approved the final submitted version of the manuscript.

### Conflict of Interest Statement

The authors declare that the research was conducted in the absence of any commercial or financial relationships that could be construed as a potential conflict of interest.
