# Supplementary material for: Assessing Executive Function in Adolescence: A Scoping Review of Existing Measures and Their Psychometric Robustness
Source: Front Psychol. 2019 Mar 1;10:311. doi: 10.3389/fpsyg.2019.00311 (PMC6405510; doi:10.3389/fpsyg.2019.00311)
Supplement: Supplementary file 1 [file Table_1.docx]

**Appendix 1: Search terms and combination**

| **Key search term** | **Synonyms** |
| --- | --- |
| Executive function* | executive control OR cognitive control OR executive functioning OR executive functioning skill OR executive functioning skills OR high order executive function OR high order executive functions OR higher order executive function OR higher order executive functions OR higher order executive functioning |
| **AND** |  |
| Adolescen* | child OR children OR young people OR teen* OR youth* |
| **AND** |  |
| measure* | tool* OR test* OR instrument* |
