# Supplementary material for: Assessing Executive Function in Adolescence: A Scoping Review of Existing Measures and Their Psychometric Robustness
Source: Front Psychol. 2019 Mar 1;10:311. doi: 10.3389/fpsyg.2019.00311 (PMC6405510; doi:10.3389/fpsyg.2019.00311)
Supplement: Supplementary file 2 [file Table_2.docx]

**Appendix 2: List of all the identified measures of EF, n=338**

|  | **Executive Function measure** | **Frequency Count** | **% frequency** |
| --- | --- | --- | --- |
| 1 | Digit Span (forward and/or backward) | 160 | 6.87 |
| 2 | Trail Making Tests (TMT) -A and/or B | 158 | 6.79 |
| 3 | **Behaviour Rating Inventory of Executive Function (BRIEF)** | 148 | 6.36 |
| 4 | Wisconsin Card Sorting Test (WCST) | 140 | 6.01 |
| 5 | Verbal fluency test | 88 | 3.78 |
| 6 | Stroop Color Word Test (SCWT) | 78 | 3.35 |
| 7 | Original Stroop task/test | 63 | 2.71 |
| 8 | Rey-Osterrieth Complex Figure test (ROCFT) | 62 | 2.66 |
| 9 | DKEFS Color-word interference test (CWIT) | 62 | 2.66 |
| 10 | CPT original (Continuous Performance Test) | 58 | 2.49 |
| 11 | Controlled Oral Word Association Test (COWAT) | 51 | 2.19 |
| 12 | Spatial span task | 46 | 1.98 |
| 13 | Go/No-go [classical] | 45 | 1.93 |
| 14 | DKEFS Tower test | 43 | 1.85 |
| 15 | Tower of London (TOL) | 41 | 1.76 |
| 16 | Conner's CPT | 38 | 1.63 |
| 17 | Stop-signal task (SST) | 38 | 1.63 |
| 18 | SWM (Spatial Working Memory) task | 32 | 1.37 |
| 19 | Letter-Number Sequencing | 30 | 1.29 |
| 20 | Intradimensional/Extradimensional task (ID/ED) | 28 | 1.20 |
| 21 | Design Fluency test | 26 | 1.12 |
| 22 | DKEFS Sorting test | 23 | 0.99 |
| 23 | SOC (Stockings of Cambridge) | 22 | 0.95 |
| 24 | Eriksen Flanker Task | 19 | 0.82 |
| 25 | Rapid Visual information Processing (RVP) | 18 | 0.77 |
| 26 | Antisaccade Task | 15 | 0.64 |
| 27 | Block Design test | 15 | 0.64 |
| 28 | Symbol search task | 14 | 0.60 |
| 29 | The Iowa Gambling Task | 14 | 0.60 |
| 30 | N-back test (verbal & visual ) | 13 | 0.56 |
| 31 | Symbol Digit Modalities Test (SDMT) | 13 | 0.56 |
| 32 | Task-Switching Test (TST) | 12 | 0.52 |
| 33 | Coding task | 12 | 0.52 |
| 34 | Porteus Mazes | 12 | 0.52 |
| 35 | The Attention Network (ANT) test | 12 | 0.52 |
| 36 | 20 Questions Task (20Q) | 11 | 0.47 |
| 37 | Digit Symbol Coding | 11 | 0.47 |
| 38 | Self-Ordered Pointing Task (SOPT) | 10 | 0.43 |
| 39 | Tower of Hanoi (ToH) | 9 | 0.39 |
| 40 | Symbol digit | 9 | 0.39 |
| 41 | Visuo-spatial [dual / double] Working Memory Task (VWMT) | 9 | 0.39 |
| 42 | TEA-Ch Score! | 9 | 0.39 |
| 43 | Matrix reasoning task | 8 | 0.34 |
| 44 | TEA-Ch Sky Search (SS) | 8 | 0.34 |
| 45 | d2-Concentration-Test (or d2 test of attention) | 8 | 0.34 |
| 46 | Colour Trails Test (1 &/or 2) | 7 | 0.30 |
| 47 | Affective Go, No-Go (AGN) | 7 | 0.30 |
| 48 | Word context test | 7 | 0.30 |
| 49 | Number-letter switching task | 7 | 0.30 |
| 50 | The Keep track Task (KTT) | 7 | 0.30 |
| 51 | Letter-memory task | 7 | 0.30 |
| 52 | Reaction Time Index (RTI) | 7 | 0.30 |
| 53 | Corsi Block [Tapping] Test (CBT) | 7 | 0.30 |
| 54 | Choice reaction time (CRT) test | 7 | 0.30 |
| 55 | Simple reaction time (SRT) test | 7 | 0.30 |
| 56 | **The Behavior Assessment System for Children (BASC-2)** | 6 | 0.26 |
| 57 | Category-switch task | 6 | 0.26 |
| 58 | Color shape task | 6 | 0.26 |
| 59 | Switching of Attention Test [SOAT] | 6 | 0.26 |
| 60 | Contingency Naming Test (CNT) | 6 | 0.26 |
| 61 | BADS-C Key Search test | 6 | 0.26 |
| 62 | BADS-C Zoo Map test | 6 | 0.26 |
| 63 | Hayling Sentence Completion Test (HSCT) | 6 | 0.26 |
| 64 | Working Memory Test Battery for Children (WMTB-C) listening recall subset | 6 | 0.26 |
| 65 | Five-Point Test | 5 | 0.21 |
| 66 | Saccade control task (prosaccade) | 5 | 0.21 |
| 67 | Test of Variables of Attention (TOVA) | 5 | 0.21 |
| 68 | Children’s Memory Scale (CMS) | 5 | 0.21 |
| 69 | Comprehensive Trail Making Test (CTMT) | 4 | 0.17 |
| 70 | Spatial 2-back task | 4 | 0.17 |
| 71 | Working Memory Index (WMI) | 4 | 0.17 |
| 72 | Prepotent Response Inhibition task | 4 | 0.17 |
| 73 | Simon's perceptual task (SP task) | 4 | 0.17 |
| 74 | Spatial delayed response task | 4 | 0.17 |
| 75 | Verbal dual [double] task | 4 | 0.17 |
| 76 | BADS-C Playing Cards [Rule Shift Cards] test | 4 | 0.17 |
| 77 | BAD-C six part [six element] test | 4 | 0.17 |
| 78 | TEA-Ch Sky Search Dual Task (SS DT) | 4 | 0.17 |
| 79 | TEA-Ch The Creature Counting (CC) | 4 | 0.17 |
| 80 | TEA-Ch Walk! Don’t Walk task | 4 | 0.17 |
| 81 | Baseline Speed task | 4 | 0.17 |
| 82 | Shifting attentional set task | 4 | 0.17 |
| 83 | Hayling & Brixton tests | 4 | 0.17 |
| 84 | Hayling subtest of Hayling & Brixton test | 4 | 0.17 |
| 85 | WMTB-C digit recall (forward & backward) | 4 | 0.17 |
| 86 | Immediate Memory Task and Delayed Memory Task (IMT/DMT) | 3 | 0.13 |
| 87 | Visual Matching test | 3 | 0.13 |
| 88 | The inspection time (IT) task | 3 | 0.13 |
| 89 | Letter N-back test | 3 | 0.13 |
| 90 | Letter 2-Back | 3 | 0.13 |
| 91 | Knock and Tap | 3 | 0.13 |
| 92 | Mexican Pyramid [pyramid of Mexico] | 3 | 0.13 |
| 93 | Dots-Triangles task | 3 | 0.13 |
| 94 | Visual span test | 3 | 0.13 |
| 95 | Count span task | 3 | 0.13 |
| 96 | Word [Problem] span task | 3 | 0.13 |
| 97 | Letter digit span [LDS] task | 3 | 0.13 |
| 98 | Category Listening Span (CLS) task | 3 | 0.13 |
| 99 | BADS-C (for 7-16yr olds) | 3 | 0.13 |
| 100 | Oddball task | 3 | 0.13 |
| 101 | TEA-Ch Code Transmission | 3 | 0.13 |
| 102 | Feature Identification [intergration] test | 3 | 0.13 |
| 103 | Memory Search Letters [two-dimensional objects] | 3 | 0.13 |
| 104 | Cognitive Assessment System (CAS) | 3 | 0.13 |
| 105 | CAS planned codes | 3 | 0.13 |
| 106 | CAS planned connections | 3 | 0.13 |
| 107 | CAS Number Detection | 3 | 0.13 |
| 108 | CAS Receptive Attention | 3 | 0.13 |
| 109 | Verbal Interference task [VIT] | 3 | 0.13 |
| 110 | Finger Windows Test | 3 | 0.13 |
| 111 | Knox-Cube Test | 3 | 0.13 |
| 112 | CogState GMLT (Groton Maze Learning Test) | 3 | 0.13 |
| 113 | CogState GMLT-Delayed Recall | 3 | 0.13 |
| 114 | PASAT (Paced Auditory Serial Addition Task) | 3 | 0.13 |
| 115 | Odd-One-Out test | 3 | 0.13 |
| 116 | The Card Playing Task | 2 | 0.09 |
| 117 | Integrated Visual and Auditory Continuous Performance Test (IVA) | 2 | 0.09 |
| 118 | Children’s Color Trails Test (CCTT) | 2 | 0.09 |
| 119 | **Diabetes Related Executive Functioning Scale (DREFS) -PR** | 2 | 0.09 |
| 120 | Emotional Stroop task | 2 | 0.09 |
| 121 | Number-quantity Stroop | 2 | 0.09 |
| 122 | Chimeric animal Stroop | 2 | 0.09 |
| 123 | Counting Stroop task | 2 | 0.09 |
| 124 | Stroop residual [interference] test | 2 | 0.09 |
| 125 | Ruff Figural Fluency Test (RFFT) | 2 | 0.09 |
| 126 | Five-Digit test (FDT) | 2 | 0.09 |
| 127 | Boston Naming Test (BNT) | 2 | 0.09 |
| 128 | Arrows Task | 2 | 0.09 |
| 129 | Spatial n-back | 2 | 0.09 |
| 130 | Rapid Automatized Naming Test (RAN) | 2 | 0.09 |
| 131 | Processing Speed Index (PSI) | 2 | 0.09 |
| 132 | Visual Attention Test | 2 | 0.09 |
| 133 | Auditory Attention and Response Set test | 2 | 0.09 |
| 134 | Animal Sorting | 2 | 0.09 |
| 135 | Word Generation task | 2 | 0.09 |
| 136 | Speeded Naming task | 2 | 0.09 |
| 137 | Spatial memory span task | 2 | 0.09 |
| 138 | Big/ Little Circle | 2 | 0.09 |
| 139 | Recognition span task | 2 | 0.09 |
| 140 | Reading Span task | 2 | 0.09 |
| 141 | Letter number span | 2 | 0.09 |
| 142 | Operation span task | 2 | 0.09 |
| 143 | Non-spatial WM tasks | 2 | 0.09 |
| 144 | BADS-A (Behavioural Assessment of the Dysexecutive Syndrome) | 2 | 0.09 |
| 145 | BADS-C water test | 2 | 0.09 |
| 146 | BAD-C the action program test | 2 | 0.09 |
| 147 | BADS-C temporal judgement test | 2 | 0.09 |
| 148 | **DEX (Dysexecutive Questionnaire (for Children, DEX-C)** | 2 | 0.09 |
| 149 | Self-Ordered Search-Verbal (SOS-V) | 2 | 0.09 |
| 150 | Self-Ordered Search-Object (SOS-O) | 2 | 0.09 |
| 151 | TEA-Ch Score-DT | 2 | 0.09 |
| 152 | TEA-Ch telephone search task | 2 | 0.09 |
| 153 | TEA-Ch telephone task while counting | 2 | 0.09 |
| 154 | TEA-Ch opposite worlds task | 2 | 0.09 |
| 155 | Psychomotor Vigilance Task (PVT) | 2 | 0.09 |
| 156 | Organization Arrows task | 2 | 0.09 |
| 157 | Brixton Spatial anticipation test | 2 | 0.09 |
| 158 | CAS Matching Numbers | 2 | 0.09 |
| 159 | CAS Expressive Attention | 2 | 0.09 |
| 160 | Buschke's selective reminding task | 2 | 0.09 |
| 161 | Balloon Analogue Risk Task (BART) | 2 | 0.09 |
| 162 | Conditional Exclusion Test | 2 | 0.09 |
| 163 | Counting Interference Test (CIT) | 2 | 0.09 |
| 164 | Local-global task | 2 | 0.09 |
| 165 | Benton Visual retention test (BVRT) | 2 | 0.09 |
| 166 | Letter Cancellation Task | 2 | 0.09 |
| 167 | CogState Set shifting task | 2 | 0.09 |
| 168 | Proactive Interference (PI) task | 2 | 0.09 |
| 169 | WMTB-C Block recall | 2 | 0.09 |
| 170 | Random Number Generation task (RNGT) | 2 | 0.09 |
| 171 | Running Memory task | 2 | 0.09 |
| 172 | Theory of Mind (TOM) subtest of the NEPSY-II | 2 | 0.09 |
| 173 | Ruff 2-&-7 Selective Attention Test | 2 | 0.09 |
| 174 | Sustained Attention to Response Task (SART) | 2 | 0.09 |
| 175 | Letter Digit Substitution Test (LDST) | 2 | 0.09 |
| 176 | Digit Symbol Substitution Test (DSST) | 2 | 0.09 |
| 177 | Swanson Sentence Span Task (SSST) | 2 | 0.09 |
| 178 | Dimensional Change Card Sort Test (DCCS) | 1 | 0.04 |
| 179 | Weigl Color Form Sort Test (WEIGL) | 1 | 0.04 |
| 180 | List Sorting Working Memory Test | 1 | 0.04 |
| 181 | The Delay of Gratification Task (DGT) | 1 | 0.04 |
| 182 | Seidman Continuous Performance Test vigilance | 1 | 0.04 |
| 183 | Taylor Complex Figure Test (TCFT) | 1 | 0.04 |
| 184 | Wisconsin Monster Sorting Test | 1 | 0.04 |
| 185 | Madrid Card-Sorting Test (MCST) | 1 | 0.04 |
| 186 | **Ballet Executive Scale (BES)** | 1 | 0.04 |
| 187 | **Behaviour Dyscontrol Scale (BDS-2)** | 1 | 0.04 |
| 188 | Stroop Match-to-Sample Task | 1 | 0.04 |
| 189 | ClinicaVR: Classroom-Stroop | 1 | 0.04 |
| 190 | Motor Stroop task | 1 | 0.04 |
| 191 | Controlled Animal Fluency Test (CAFT) | 1 | 0.04 |
| 192 | Retrieval Fluency | 1 | 0.04 |
| 193 | Regensburger Wortflüssigkeits test | 1 | 0.04 |
| 194 | Tower of Coimbra | 1 | 0.04 |
| 195 | Number-pinyin task | 1 | 0.04 |
| 196 | Strategic Learning Task (SLT) | 1 | 0.04 |
| 197 | Penn Short Letter N-Back Test (SLNB) | 1 | 0.04 |
| 198 | Object 2-back | 1 | 0.04 |
| 199 | 2-n-back task | 1 | 0.04 |
| 200 | One back paradigm | 1 | 0.04 |
| 201 | Naming letters task | 1 | 0.04 |
| 202 | Selective Auditory Attention Task | 1 | 0.04 |
| 203 | The Clocks test | 1 | 0.04 |
| 204 | Phonological Processing | 1 | 0.04 |
| 205 | Statue | 1 | 0.04 |
| 206 | Design Copying | 1 | 0.04 |
| 207 | Executive Maze Task [EM] | 1 | 0.04 |
| 208 | Virtual Water Maze | 1 | 0.04 |
| 209 | Reasoning and Problem-Solving mazes | 1 | 0.04 |
| 210 | Arena Maze | 1 | 0.04 |
| 211 | Simon's spatial interference task | 1 | 0.04 |
| 212 | Flanker Fish Tasks (FF) | 1 | 0.04 |
| 213 | Flanker Inhibitory Control and Attention Test | 1 | 0.04 |
| 214 | Flanker Shape (FS) | 1 | 0.04 |
| 215 | Flanker visual filtering task | 1 | 0.04 |
| 216 | Booklet Category Test(BCT) | 1 | 0.04 |
| 217 | Children’s Category Test | 1 | 0.04 |
| 218 | Children's Cooking Task (CCT) | 1 | 0.04 |
| 219 | Children’s Checking Task | 1 | 0.04 |
| 220 | Children’s size-ordering task | 1 | 0.04 |
| 221 | Matrix Span Task (MST) | 1 | 0.04 |
| 222 | Computation span task | 1 | 0.04 |
| 223 | Selective span task | 1 | 0.04 |
| 224 | Running span task | 1 | 0.04 |
| 225 | Spatial self-ordered search task | 1 | 0.04 |
| 226 | BADS-C Similarities Task | 1 | 0.04 |
| 227 | Luria Hand Game | 1 | 0.04 |
| 228 | Catch Game test | 1 | 0.04 |
| 229 | Planning drawing task, part B | 1 | 0.04 |
| 230 | **Dynamic Occupation Assessment of Executive Function (DOAEF)** | 1 | 0.04 |
| 231 | **Amsterdam executive function inventory (AEFI)** | 1 | 0.04 |
| 232 | **Cognitive Abilities Questionnaire (CAQ)** | 1 | 0.04 |
| 233 | TEA-Ch map mission task | 1 | 0.04 |
| 234 | Batteria di valutazione neuropsicologica (BVN) test of attention | 1 | 0.04 |
| 235 | Bells test | 1 | 0.04 |
| 236 | Nonword Repetition task (NWR) | 1 | 0.04 |
| 237 | Number repetition backwards of the Clinical Evaluation of Language Fundamentals (CELF) | 1 | 0.04 |
| 238 | Encoding task | 1 | 0.04 |
| 239 | Visual Spatial Sequencing (VSS) task | 1 | 0.04 |
| 240 | Sustained attention dots task | 1 | 0.04 |
| 241 | Sustained attention auditory task | 1 | 0.04 |
| 242 | Choice delay task | 1 | 0.04 |
| 243 | Serial subtractions | 1 | 0.04 |
| 244 | Serial digit learning | 1 | 0.04 |
| 245 | Risky Choice Task (RCT) | 1 | 0.04 |
| 246 | Columbia Card Task (CCT) | 1 | 0.04 |
| 247 | **Decision Making Quality Scale** | 1 | 0.04 |
| 248 | Decision making task | 1 | 0.04 |
| 249 | Cambridge Gambling Task | 1 | 0.04 |
| 250 | **BIS/BAS (Behavioral Inhibition System /Behavioural Activation System)** | 1 | 0.04 |
| 251 | Frontal Screening test (IFS) | 1 | 0.04 |
| 252 | **Shipley Institute of Living Scale** | 1 | 0.04 |
| 253 | Rorschach cognitive-perceptual problem-solving task | 1 | 0.04 |
| 254 | Problem Solving test | 1 | 0.04 |
| 255 | EpiTrack Junior | 1 | 0.04 |
| 256 | Time estimation task | 1 | 0.04 |
| 257 | Repeatable Battery for the Assessment of neuropsychological Status (RBANS) | 1 | 0.04 |
| 258 | Signs Cancellation Tests (2 and 3) | 1 | 0.04 |
| 259 | Pair Cancellation test | 1 | 0.04 |
| 260 | Color Cancelation Test | 1 | 0.04 |
| 261 | Number Cancellation test | 1 | 0.04 |
| 262 | Bell Cancellation Task | 1 | 0.04 |
| 263 | Dot cancellation task | 1 | 0.04 |
| 264 | Cancel Underline (CUL) | 1 | 0.04 |
| 265 | The Opposite Emotions Test (OET) | 1 | 0.04 |
| 266 | Stimulus-response compatibility task | 1 | 0.04 |
| 267 | Preparing to Overcome Prepotency (POP) task | 1 | 0.04 |
| 268 | Plus minus test | 1 | 0.04 |
| 269 | Dichotic Listening (DL) task | 1 | 0.04 |
| 270 | T-P Perception and Attention Test | 1 | 0.04 |
| 271 | A calibration block task | 1 | 0.04 |
| 272 | Progressive planning test | 1 | 0.04 |
| 273 | Revised Strategy Application Test (R-SAT) | 1 | 0.04 |
| 274 | Bourdon–Vos test (a correction test) | 1 | 0.04 |
| 275 | Tasks of Executive Control (TEC) | 1 | 0.04 |
| 276 | Weekly Calendar Planning Activity (WCPA) | 1 | 0.04 |
| 277 | Verbal production task | 1 | 0.04 |
| 278 | Categorization task | 1 | 0.04 |
| 279 | CogState Groton Maze Chase Test (GMCT) | 1 | 0.04 |
| 280 | CogState Detection task | 1 | 0.04 |
| 281 | CogState Identification task | 1 | 0.04 |
| 282 | Matching Familiar Figures Test (MFFT) | 1 | 0.04 |
| 283 | Brown-Peterson test. | 1 | 0.04 |
| 284 | The Freedom from Distractibility Index | 1 | 0.04 |
| 285 | Multi-Source Interference Task | 1 | 0.04 |
| 286 | Eyes Looking (EL) task | 1 | 0.04 |
| 287 | Heart and Flowers (HF) Task | 1 | 0.04 |
| 288 | Stanford-Binet memory for digits | 1 | 0.04 |
| 289 | NEUPSILIN battery test | 1 | 0.04 |
| 290 | Staged Information Processing Speed test | 1 | 0.04 |
| 291 | Brief Test of Attention | 1 | 0.04 |
| 292 | Test of Attentional Performance (TAP) | 1 | 0.04 |
| 293 | AUT (Alternative Uses Task) | 1 | 0.04 |
| 294 | WMTB-C Word List Matching | 1 | 0.04 |
| 295 | WMTB-C Word List Recall | 1 | 0.04 |
| 296 | WMTB-C Non-Word List Recall | 1 | 0.04 |
| 297 | WMTB-C Counting Recall | 1 | 0.04 |
| 298 | WMTB-C Mazes Memory | 1 | 0.04 |
| 299 | **Working Memory Rating Scale** | 1 | 0.04 |
| 300 | Verbal inhibition task doll/car | 1 | 0.04 |
| 301 | Verbal Inhibition/Motor Inhibition (VIMI) task | 1 | 0.04 |
| 302 | GoStop Impulsivity Paradigm (GoStop) | 1 | 0.04 |
| 303 | Two Choice Impulsivity Paradigm (TCIP) | 1 | 0.04 |
| 304 | Single Key Impulsivity Paradigm (SKIP) | 1 | 0.04 |
| 305 | The Hungry Donkey Task (HDT) | 1 | 0.04 |
| 306 | Tic Tac Toe | 1 | 0.04 |
| 307 | The Mental Counters task | 1 | 0.04 |
| 308 | Smiling Faces task | 1 | 0.04 |
| 309 | Director task | 1 | 0.04 |
| 310 | Dot probe task | 1 | 0.04 |
| 311 | Real Animal Size Test | 1 | 0.04 |
| 312 | Pictorial Animal Size Test | 1 | 0.04 |
| 313 | Executive Golf Task | 1 | 0.04 |
| 314 | Baddeley Working Memory Task | 1 | 0.04 |
| 315 | Pathways task | 1 | 0.04 |
| 316 | Starting position selection task | 1 | 0.04 |
| 317 | Fragmented Pictures Task (FPT) | 1 | 0.04 |
| 318 | Alternating runs | 1 | 0.04 |
| 319 | The Dot matrix | 1 | 0.04 |
| 320 | Posner task | 1 | 0.04 |
| 321 | Symbol distance effect (SDE) task | 1 | 0.04 |
| 322 | Memory scanning task | 1 | 0.04 |
| 323 | Backward Masking Test (BMT) | 1 | 0.04 |
| 324 | Span of Apprehension (SPAN) task | 1 | 0.04 |
| 325 | Spatial-temporal working memory task | 1 | 0.04 |
| 326 | Rhyming words working memory task | 1 | 0.04 |
| 327 | Visual matrix working memory task | 1 | 0.04 |
| 328 | Mapping and directions task | 1 | 0.04 |
| 329 | Semantic association task | 1 | 0.04 |
| 330 | Semantic categorization task | 1 | 0.04 |
| 331 | The Identical Pictures Test | 1 | 0.04 |
| 332 | Digit vigilance test | 1 | 0.04 |
| 333 | Vigilance task (from Gordon Diagnostic system) | 1 | 0.04 |
| 334 | Triads task from NIMHANS neuropsychological battery | 1 | 0.04 |
| 335 | Shape Matching (SM) task | 1 | 0.04 |
| 336 | Concept Shifting Test | 1 | 0.04 |
| 337 | Digit Running Test (DRT) | 1 | 0.04 |
| 338 | Distractibility task (from Gordon Diagnostic system) | 1 | 0.04 |
|  | **Total frequency** | 2328 | 100% |
| Bolded are rating scales (n=13).  **DKEFS**, Delis-Kaplan Executive Function System | | | |
