# Supplementary material for: Assessing Executive Function in Adolescence: A Scoping Review of Existing Measures and Their Psychometric Robustness
Source: Front Psychol. 2019 Mar 1;10:311. doi: 10.3389/fpsyg.2019.00311 (PMC6405510; doi:10.3389/fpsyg.2019.00311)
Supplement: Supplementary file 3 [file Table_3.docx]

**Appendix 3: Summary description of the 10 frequently used individual measures of EF**

| **Name of Measure of EF** | **Mode of administration** | **Time taken to administer** | **Age target** | **Description of domain assessed and summary of administration procedure** |
| --- | --- | --- | --- | --- |
| BRIEF ([Gioia et al., 2000](#_ENREF_36); [Guy et al., 2004](#_ENREF_41)) | Informant or  self-report | 10 - 15 minutes | 5 to 18 years (informant) 11 to 18 years (self-report) | **Domain:** ***Behavioral Regulation Index*** (Inhibition, Shift, Emotional Control, Initiation sub-scales); **Meta cognition Index** (Working Memory, Planning, Organization of Materials and Monitoring sub-scales)  **Procedure:** Informant/participant is asked questions that evaluate the cognitive, emotional and behavioural function of the subject under the above 8 clinical domains |
| Classical (original) Stroop Task* ([Stroop, 1935](#_ENREF_92)) | Test taking | About 5 to 8 minutes (timed) | 6 to 80 years | **Domain:** Resistance to interference and response inhibition  **Procedure**: Includes 3 cards (word card; colour card; and colour-word card), *5 colours are used* (red, blue, green, brown, purple).  1) Word card – a subject is asked to name 100 random words for colours printed on a sheet of paper in black ink as quickly as she/he can within 45 seconds.  2) Colour card – a subject is asked to read 100 random words printed in colours that are congruent with the words (e.g. to read RED printed in red colour) as fast as she/he can within 45 seconds.  3) Colour-word card – 100 random words are printed in incongruent colours (e.g. the word RED might be printed in “brown” or “green” colour). The subject is required to ignore the word and state the colour of the ink as fast as she/he can within 45 seconds. |
| Continuous Performance Test^$^ ([Rosvold et al., 1956](#_ENREF_86)) | Test taking | About 15 minutes | Children and adults (8 years and over) | **Domain:** Sustained and selective attention  **Procedure:** A participant is shown a series of letters. In the first part of the experiment, she/he must respond whenever an "X" is displayed. In the second part, she/he must respond whenever "A" followed by an "X" is displayed. |
| Digit Span ([Blackburn and Benton, 1957](#_ENREF_11)) | Test taking | 2 - 5 minutes | Children and adults (13–98 years) | **Domain:** Verbal and spatial working memory.  **Procedure:** An individual repeats sequence of numbers in the same and/or reverse order as presented. The digit length progresses gradually in terms of level of difficulty. |
| D-KEFS Colour-Word Interference Test* ([Delis et al., 2001](#_ENREF_27)) | Test taking | 5 minutes (timed) | 8 to 89 years | **Domain:** Cognitive flexibility, attention, resistance to interference and response inhibition  **Procedure**: Includes 4 trial conditions (colour naming, word reading, inhibition and inhibition/switching), *3 colours are used* (red, green and blue).  1) Colour naming – a participant is presented with a series of red, green and blue squares on a sheet of paper and is asked to say the names of the colours as quickly as she/he can.  2) Word reading – a subject is asked to read a series of random words for colours printed on a sheet of paper in black ink as quickly as she/he can.  3) Inhibition – Similar to the Stroop Colour-Word card. A participant is presented with a series of words (red, green or blue) printed incongruently either in red, green, or blue ink. She/he is asked to say the colour of the ink in which each word is printed (ignoring the word) as quickly as she/he can.  4) Inhibition/switching – similar to inhibition only that additionally, half of the words printed in incongruent colours are enclosed within rectangular boxes. For these, a participant is required to switch to naming the word instead of the ink colour. |
| Rey-Osterreith Complex Figure Test ([Rey, 1941](#_ENREF_83); [Osterrieth, 1944](#_ENREF_68)) | Test taking | About 45 minutes (3 and 30 min timed recall) | 6 to 89 years | **Domain:** Planning, organizational skills, problem solving, perceptual and memory abilities.  **Procedure:** An individual is asked to copy a figure (with stimulus card present). Later, at two separate time points (3 and 30 minutes delay), she/he is again asked to reproduce the figure from memory. A recognition portion then follows where participants circle the appropriate portions of the figure. |
| Stroop Colour-Word Test* ([Golden and Freshwater, 1978](#_ENREF_37)) | Test taking | 5 minutes (timed) | 5 to 14 (child version)  15 to 90 years (adult version) | **Domain:** Cognitive flexibility, attention, resistance to interference and response inhibition  **Procedure**: Includes 3 cards (word card; colour card; and colour-word card), *3 colours are used* (red, green and blue).  1) Word card – a subject is asked to name 100 random words for colours printed on a sheet of paper in black ink as quickly as they can within 45 seconds  2) Colour card – a subject is asked to read 100 random words printed in colours that are congruent with the words (e.g. to read RED printed in red colour) as fast as they can within 45 seconds  3) Colour-word card – 100 random words are printed in incongruent colours (e.g. the word RED might be printed in “blue” or “green” colour). The subject is required to ignore the word and name the colour of the ink as fast as they can within 45 seconds |
| Trail Making Test ([Reitan, 1992](#_ENREF_82)) | Test taking | 5 - 12 minutes | 8 to 89 years | **Domain:** Psychomotor speed, cognitive flexibility, working memory.  **Procedure:** An individual is supposed to connect randomly arranged encircled numbers and/or letters, by making lines, in proper order and as fast as they can. |
| Verbal fluency tasks^#^ ([Benton, 1968](#_ENREF_8); [Newcombe, 1969](#_ENREF_67)) | Test taking | 1 to 4.5 minutes (depending on condition used) | 8 to 89 years | **Domain:** Orthographic knowledge, Semantic knowledge, retrieval, cognitive flexibility and set shifting  Procedure: Includes 3 conditions (Phonetic/Letter fluency, Semantic/Category fluency, and Category switching)  1) Phonetic/Letter fluency - the subject is asked to name all words that begin with a particular letter, as quickly as possible in a fixed period, typically 1 minute. The most common letters chosen are F, A, and S or C, F, and L, normally 60 seconds per letter, although 90 seconds is also used.  2) Semantic/Category fluency - requires that the subject names as many members of a semantic category as possible in a fixed period, typically 1 minute. This is usually animal naming although some variations include listing of fruits, furniture, vegetables, musical instruments or professions.  3) Category switching -requires that the subject names as many members of a semantic category as possible in a fixed period, but switching in between categories (e.g. switching between fruits/furniture) |
| Wisconsin Card Sorting Task ([Heaton, 1981](#_ENREF_43)) | Test taking | 20 - 30 minutes | 6.5 to 89 years | **Domain:** Perseveration, abstract reasoning, working memory and cognitive flexibility  **Procedure:** An individual completes a complex categorization set shifting task and/or responds to feedback from the computer. |
| **NB: D-KEFS** – Delis-Kaplan Executive Function System  ***** Several variations of Stroop’s original procedures are used within the filed. These have differed on: i) the colours used; ii) the number of colours used; iii) type of stimulus used to present  the colour patches; iv) order of reading the items (across rows or down the columns); and v) the method of scoring. For a detailed review see the review by [Jensen and Rohwer (1966](#_ENREF_48))  ^$^ The role of attention in EF has been widely debated in the literature. We include Continuous Performance Test as a measure of EF because this is how it was classed by authors.  **^#^** Has 3 conditions: Phonetic/letter fluency; Semantic/category fluency; and category switching | | | | |
