# Supplementary material for: Assessing Executive Function in Adolescence: A Scoping Review of Existing Measures and Their Psychometric Robustness
Source: Front Psychol. 2019 Mar 1;10:311. doi: 10.3389/fpsyg.2019.00311 (PMC6405510; doi:10.3389/fpsyg.2019.00311)
Supplement: Supplementary file 4 [file Table_4.docx]

**Appendix 4: Study sites for measures of EF used with adolescent sub-population by region and income ranking**

| **Continent** | **Frequency** | **% frequency** |
| --- | --- | --- |
| Multicenter (across continents) | 2 | 0.3 |
| Africa | 6 | 0.9 |
| South America | 14 | 2.0 |
| Asia | 40 | 5.7 |
| Oceania | 41 | 5.8 |
| Europe | 277 | 39.3 |
| North America | 325 | 46.1 |
| Total | 705 | 100.0 |

| **Income ranking** |  |  |
| --- | --- | --- |
| Low income country (LIC) | 2 | 0.3 |
| LMIC & HIC (multicenter) | 2 | 0.3 |
| Low-middle income country (LMIC) | 10 | 1.4 |
| Upper-middle income country (UMIC) | 24 | 3.4 |
| High income country (HIC) | 667 | 94.6 |
| Total | 705 | 100.0 |
